# An All-Dielectric Metasurface Polarimeter

**DOI:** 10.1021/acsphotonics.2c00395

**Published:** 2022-09-15

**Authors:** Yash D. Shah, Adetunmise C. Dada, James P. Grant, David R. S. Cumming, Charles Altuzarra, Thomas S. Nowack, Ashley Lyons, Matteo Clerici, Daniele Faccio

**Affiliations:** † School of Physics and Astronomy, University of Glasgow, Glasgow G12 8QQ, U.K.; ‡ Microsystems Technology Group, James Watt School of Engineering, University of Glasgow, Glasgow G12 8QQ, U.K.; § James Watt School of Engineering, University of Glasgow, Glasgow G12 8QQ, U.K.

**Keywords:** metasurface, polarimetry, Stokes parameters, anapoles, all-dielectric materials, metasurface-based polarimeters

## Abstract

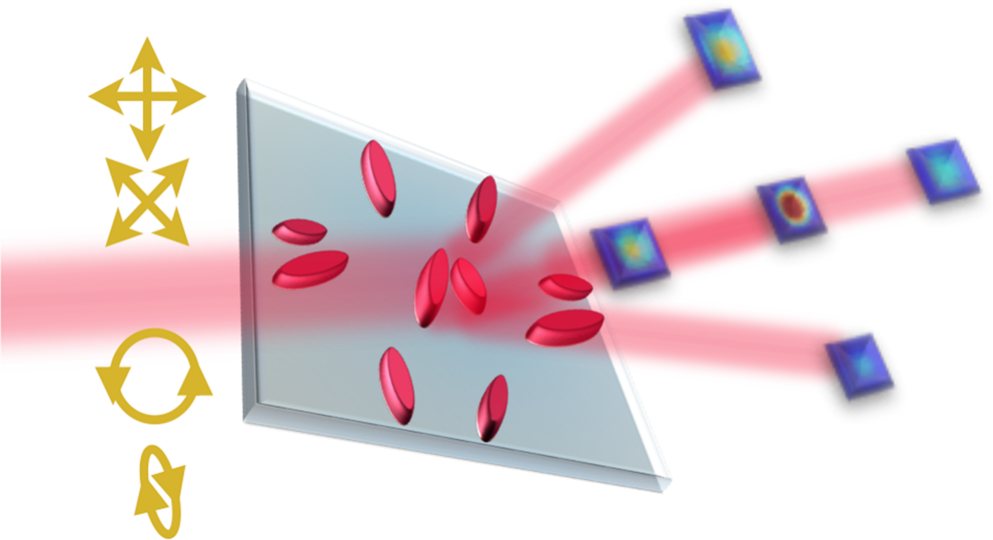

The polarization state of light is a key parameter in many imaging systems. For example, it can image mechanical stress and other physical properties that are not seen with conventional imaging and can also play a central role in quantum sensing. However, polarization is more difficult to image, and polarimetry typically involves several independent measurements with moving parts in the measurement device. Metasurfaces with interleaved designs have demonstrated sensitivity to either linear or circular/elliptical polarization states. Here, we present an all-dielectric meta-polarimeter for direct measurement of any arbitrary polarization state from a single-unit-cell design. By engineering a completely asymmetric design, we obtained a metasurface that can excite eigenmodes of the nanoresonators, thus displaying a unique diffraction pattern for not only any linear polarization state but all elliptical polarization states (and handedness) as well. The unique diffraction patterns are quantified into Stokes parameters with a resolution of 5° and with a polarization state fidelity of up to 99 ± 1%. This holds promise for applications in polarization imaging and quantum state tomography.

## Introduction

Metasurfaces or flat-optics^1,2^ are the two-dimensional counterparts of metamaterials comprising subwavelength-thickness nanostructures. The spatial distribution, geometry, and material (metallic or high-index dielectric) of these nanostructures provide dramatically enhanced light–matter interactions at subwavelength scales. This can be utilized to efficiently control and tailor local polarizations, phases, and amplitudes of linear fields.^3,4^ Metasurfaces have emerged as a promising technology for unprecedented manipulation of light, which makes them integral for nanophotonics and miniaturization of optical systems. Polarimetry, that is, the ability to measure one of the fundamental properties of light—polarization—is of significant importance from research in fundamental physics and light–matter interaction to polarization-based quantum information technology and quantum imaging.^5−7^ From polarimetry, we typically obtain the Stokes parameters, which contain all the information to represent the state of polarization (SoP) of light.^8^ While traditional polarimetry requires multiple measurements, even with moving or reconfigurable systems,^9^ metasurfaces hold promise for measurements based on static and ultra-compact components. The constituent elements that make up a metasurface (meta-atoms) can be engineered to form subwavelength-scale polarization optics with metal-based (plasmonics)^10^ or dielectric-based material systems.^11^ Polarization-sensitive metasurfaces are realized predominantly with anisotropic nanopillars (elliptical or rectangular). The orientation of these nanostructures leads to birefringence and sensitivity to the linear polarization state of light. The Pancharatnam–Berry (PB) phase or geometric phase is the accumulated phase due to the orientation and arrangement of anisotropic meta-atoms, making it sensitive to circularly polarized light. Thus, various designs have been realized for plasmonic polarimeters.^11,12^ However, a major drawback is the high absorption loss inherent in metallic structures. On the other hand, the use of dielectric-based material systems has shown improved performances with higher diffraction (and transmission) efficiencies. Forming nanostructure resonators from materials with a high refractive index contrast leads to higher scattering of electromagnetic fields, and combining this with engineering of the geometry and spatial distribution leads to higher efficiencies for SoP measurement.

For direct measurements of a polarization state, the geometry (which relates to the propagation phase) and orientation (which relates to the geometric phase) of anisotropic nanopillars are judiciously varied, providing unique designs for each of the horizontal (|*H*⟩), vertical (|*V*⟩), anti-diagonal (|*A*⟩), diagonal (|*D*⟩), left-circular (|*L*⟩) and right-circular (|*R*⟩) polarization states, which are then interleaved into one metasurface platform.^13^ While this approach could differentiate between these six degenerate polarization states, it has difficulty in measuring other polarization states, leading to spurious diffraction at the point of interlacing. By imposing arbitrary propagation and geometric phase profiles with birefringent rectangular nanopillars, designs could address elliptical polarization states as well.^14,15^ Extending this approach and using the Fourier transform of the optical field for the Jones matrix of each element, elliptical and circular polarization states were diffracted to specific spots and used for polarization-based imaging.^16^ However, in this case, a design that is sensitive to linear polarization states would have the same response to orthogonal elliptical/circular states. Metasurfaces based on silicon nanostructures that are topology-optimized using free-form design have been used to perform polarization conversion.^17^ However, this approach relies on reconfiguration of the angle of incidence to achieve the measurement of different polarization states. Holography provides a visual approach to polarimetry wherein a unique hologram needs to be encoded using the phase information of each meta-atom for each polarization state.^18^ This makes it difficult to differentiate between arbitrary polarization states. The holographic design proposed in refs^19^ and^20^ extracts the Stokes parameters, however, with a limitation in resolution as the periodicity limits how many designs can be incorporated in a single unit cell. A single-cell design that can be optimized for any desired wavelength and can successfully distinguish between not only linear and elliptical but also opposite handedness has not been realized.

Here, we introduce an all-dielectric metasurface design comprising elliptical nanopillars that, with a single-unit-cell design, provides a unique diffraction pattern for any input polarization state, covering the entire Poincaré sphere. Unlike the metasurface designs based on the PB phase, our approach relies on the control of the resonant modes supported by the nanopillar, which affects the electric and magnetic multipoles. While plasmonic metasurfaces exploit electrical resonances, dielectric nanostructures are the ideal choice as they enhance not only electrical but also magnetic resonances. Our asymmetric design exploits exotic multipolar coupling by exciting otherwise symmetry-protected eigenstates, which makes the proposed design sensitive to not only linear but circular/elliptical polarized light as well.

**Figure 1:**
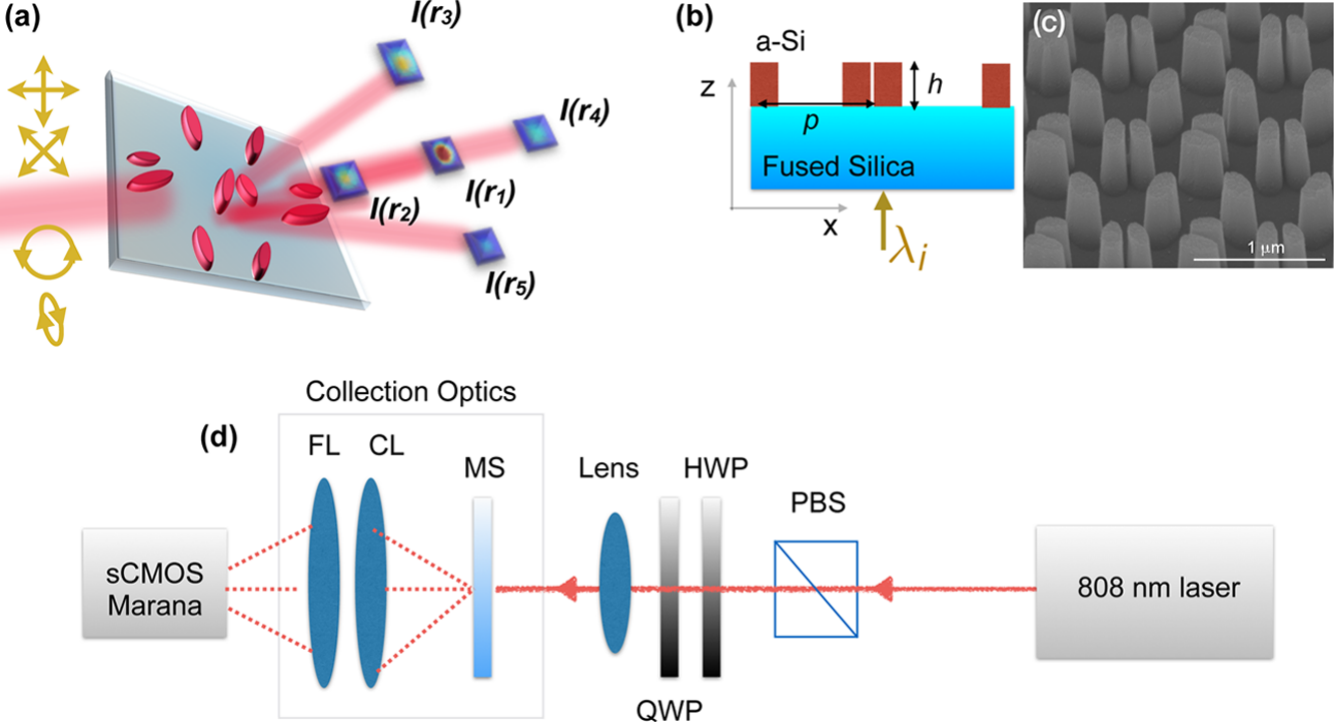
(a) Schematic of the proposed meta-polarimeter, which gives a diffraction pattern unique to any arbitrary incident polarization state. *I*(*r*_*n*_) is the intensity at each diffracted spot *n*. (b) Schematic of the cross section with light (λ_i_ = 808 nm) incident from the substrate. (c) SEM image of the fabricated metasurface. (d) Schematic of the measurement setup to obtain a single-shot diffraction pattern on a Andor Marana sCMOS camera. PBS: Polarizing beam splitter. HWP: half-wave plate. QWP: quarter-wave plate. MS: metasurface. CL: condenser lens. FL: Fresnel lens.

Our design is based on pairs of elliptical pillars, which are combined to form the unit cells of the metasurface, which are referred to here as *bi-meta-atoms*. These are arranged such that for any input polarization of light with wavelength λ_i_ ≈ 810 nm normally incident on the metasurface, we obtain five diffraction spots of varying intensity *I*(*r*_*n*_) with a unique distribution depending on the polarization state, as shown in Figure 1a. Light is incident normally on the fused-silica substrate and the amorphous-silicon (α-Si) elliptical nanopillars, see Figure 1b. The SEM image of the metasurface is shown in Figure 1c. A period *p* = 600 nm with *p* > 2λ/μ (where μ is the refractive index of the elliptical pillars) excites diffraction modes of orders ±1, 0 at an angle Γ ≈ sin^–1^(λ/*p*).

The design principle was based around finding a combination of values for geometric parameters, including the orientation of the elliptical pillars and height (*h*), for which we obtain not only the largest difference in the detected intensity distribution between |*L*⟩ and |*R*⟩ states but also high transmission (thereby higher diffraction efficiency).

The design process follows the simple principle of choosing the geometry and orientation of the structures in the bi-meta-atom for which the difference in the diffraction pattern between |*L*⟩ and |*R*⟩ states, Δ_*LR*_, is largest. We note that due to limitation of the e-beam (used in the fabrication), the minimum gap between structures in the bi-meta-atom had to be greater than 50 nm. Reference^21^ provides further insights regarding limitations on the gaps in a meta-atom arrangement. Figure S1 in the Supporting Information provides further details on the bi-meta-atoms with regard to asymmetry and tilt angles, as obtained through finite-difference time-domain (FDTD) simulations. The period between the meta-atoms is greater than λ/2*n*, such that clear diffraction spots can be observed as well as a large enough Δ_*LR*_. A possibility of using genetic algorithms to optimize the gap between the meta atoms and the ratio of geometry to height would likely provide a more optimal design in terms of diffraction efficiency (higher intensity in the spots) and pattern difference Δ_*LR*_.

**Figure 2:**
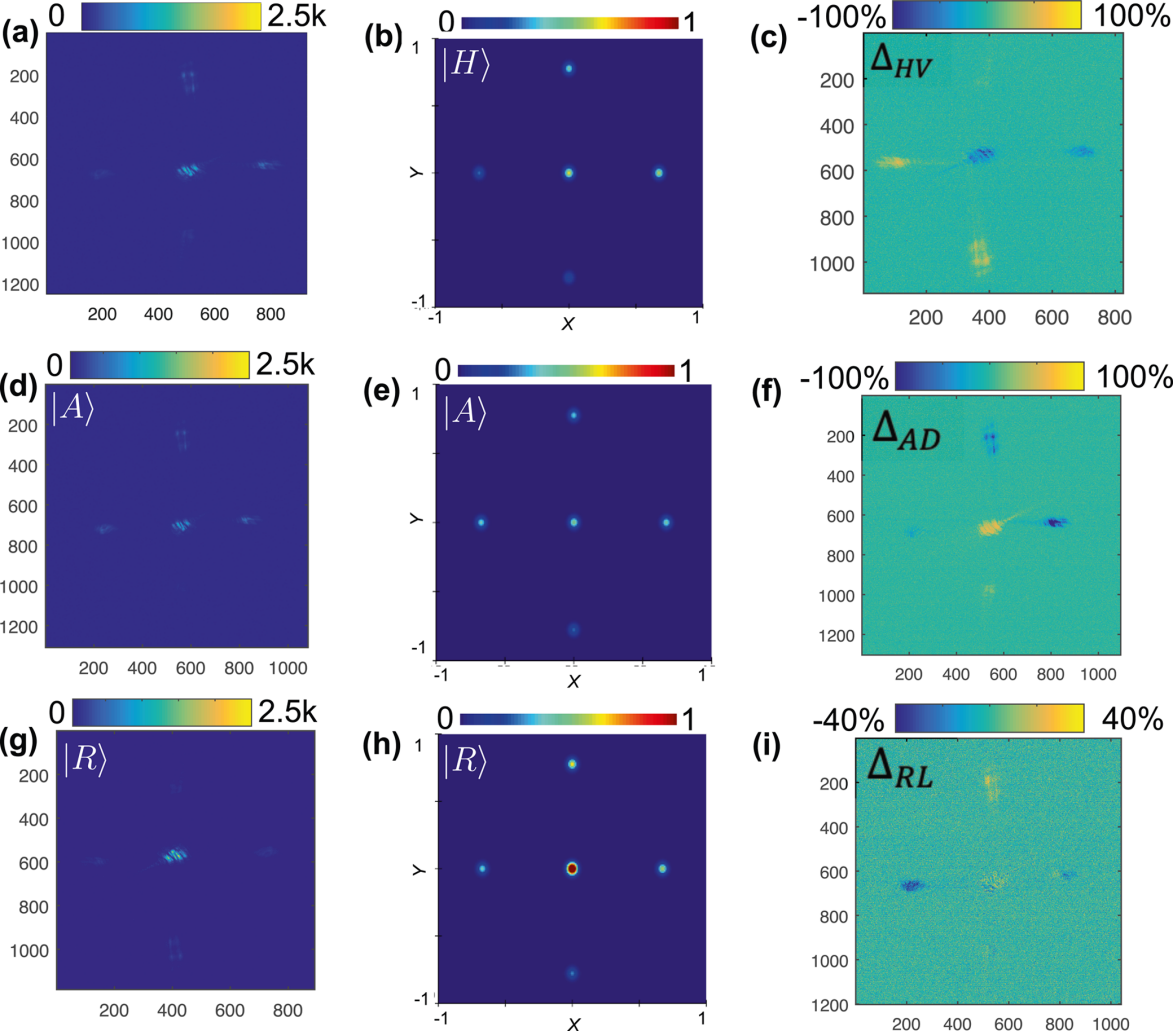
Experimental diffraction patterns and FDTD simulations for the orthogonal polarization states. The experimental diffraction spots are plotted with the scale indicating the counts, and the simulated results are plotted with the scale normalized to |*E*|^2^ in the far field for the (a,b) |*H*⟩ polarization state, the (d,e) |*A*⟩ polarization state, and the (g,h) |*R*⟩ polarization state, respectively. To highlight the differences between intensity patterns for orthogonal states, (c,f,i) show the % difference in the intensities of the diffracted spots between |*H*⟩ and |*V*⟩ (Δ_*HV*_), |*A*⟩ and |*D*⟩ (Δ_*AD*_), and |*R*⟩ and |*L*⟩ (Δ_*RL*_), respectively. Horizontal and vertical axes represent the respective positions within the lateral-plane image.

From FDTD simulations, the height *h* of the nanopillars was selected as 520 nm, which gave the highest transmission efficiency of up to 63.7% (see Section S1 of the Supporting Information). Extensive simulations were performed for the geometry and orientation of the elliptical pillars (see Sections S1 and S3 of the Supporting Information). A ratio of 0.5 is selected between the minor and major axes of the ellipse to accommodate resonances at different wavelengths without overlap. Post fabrication, the devices were characterized using the setup shown in Figure 1d to obtain a single-shot image of the diffraction pattern using a condenser lens (CL) and a 2 inch-diameter Fresnel lens (FL) (see Methods for details). The diffraction patterns for the six degenerate polarization states, |*H*⟩, |*V*⟩, |*A*⟩, |*D*⟩, |*R*⟩, and |*L*⟩, are measured using a complementary metal oxide semiconductor (CMOS) camera (Andor Marana), and examples are shown in Figure 2 along with the corresponding FDTD simulation results (see Methods).

## Results and Discussion

### Experimental Results

Each polarization state has a unique diffraction pattern with a different intensity, *I*(*r*_*n*_), in the spatially distributed spots, *n* = 1, ..., 5. Figure 2a,d,g shows the diffraction patterns from |*H*⟩, |*A*⟩, and |*R*⟩ polarization states, respectively, along with the simulated results in Figure 2b,e,h. We also plot the intensity difference (Δ*I*) of each spot for the orthogonal basis polarization states in Figure 2c,f,i. The stark differences showcase how the diffraction patterns allow us to distinguish not only |*H*⟩ (|*A*⟩) from |*V*⟩ (|*D*⟩) but also the handedness of circular polarization, |*R*⟩ and |*L*⟩.

**Figure 3:**
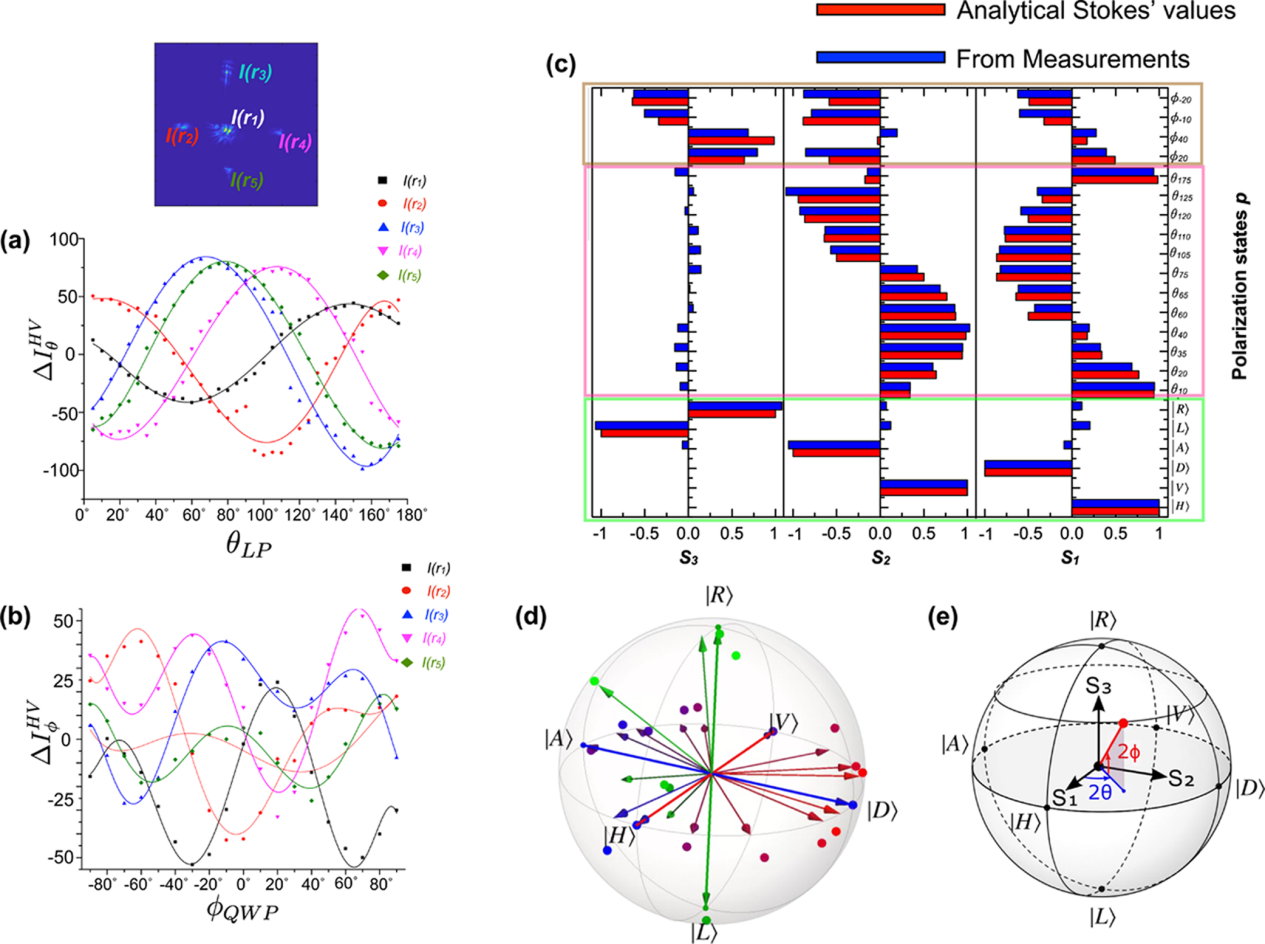
Using the intensity of the diffracted spots to extract the Stokes parameters and comparing with analytical Stokes’ values, (a) difference in intensities, Δ*I*_θ_^*HV*^, of the five diffraction spots for all linear polarization states, θ_*LP*_; (b) difference in the intensities of the five spots, Δ*I*_ϕ_^*HV*^, for all elliptical polarization states corresponding to the QWP angle ϕ_*QWP*_ = ϕ/2 with the HWP maintained at 22.5°. The dots are experimentally measured values, while the lines are polynomial fits to serve as a guide to the eye. (c) Comparison of the experimental Stokes parameters for various polarization states obtained using the metasurface matrix (blue) and analytical Stokes’ values (red). The degenerate polarization states are highlighted in green, the linear polarizations in pink, and the elliptical polarizations in brown. (d) Representation as points on the Poincaré sphere of some of the input states (arrows) and the corresponding states experimentally identified by the metasurface (large dots) from results shown in (a–c). The small dots represent the six degenerate polarization states. These measurements have an average fidelity of 99.27 ± 0.86%. (e) Illustration of the Stokes parameters’ orientations along with the azimuth and ellipticity angles, 2θ and 2ϕ, respectively.

In Figure 3a, we show , where *I*_*rn*_^θ^ is the intensity for the linear polarization input state defined by angle θ and *I*_*rn*_^*HV*^ is the sum of the respective intensities for input states |*H*⟩ and |*V*⟩ for the *n*th diffraction spot. Similarly, Figure 3b shows the plot , where *I*_*rn*_^ϕ^ is the intensity for the elliptical polarization input state defined by angle ϕ [with the half-wave plate (HWP) fixed at θ_*HWP*_ = θ/2 = 22.5° and the quarter-wave plate (QWP) at angle ϕ_*QWP*_ = ϕ/2] for the *n*th diffraction spot.

We then need to determine the Stokes parameters^22^ from these intensity patterns. From the intensity difference plots in Figure 3a, we observe that the intensity trends of the diffracted spots at positions *r*_3_ and *r*_5_ are nearly equal. In other words, we need to only consider *r*_3_ or *r*_5_ but not both. Thus, the information from the intensity trends of diffracted spots at the four positions *r*_1_, *r*_2_, *r*_4_, and *r*_5_ is used to obtain the Stokes parameters, *S*_1_, *S*_2_, and *S*_3_.

The intensity from various spots from all polarization states can be quantified by a metasurface matrix, [*M*], such that [*I*] = [*M*] × [*S*], where [*I*] is the intensity vector and [*S*] is the Stokes vector. Explicitly,
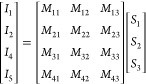
1

The intensity vector [*I*] contains the intensity differences either Δ*I*_θ_^*HV*^ or Δ*I*_ϕ_^*HV*^ and the Stokes vector [*S*] contains the Stokes parameters (*S*_1_, *S*_2_, and *S*_3_) normalized to *S*_0_. The metasurface matrix [*M*] is calculated from the intensities of the |*H*⟩, |*V*⟩, |*A*⟩, |*D*⟩, |*R*⟩, and |*L*⟩ states. We note that [*M*] embodies the linear relationship between the Stokes parameters and the elements of the intensity vector.^23^ In other words, the metasurface matrix embodies the description of how the device behaves under illumination with light of any (arbitrary) polarization since it can be used to obtain the intensity pattern corresponding to any input polarization and also to determine the polarization corresponding to any given intensity pattern.

We determined [*M*] to be
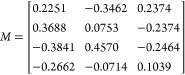
2which can be shown to be a matrix of rank 3, indicating the ability to span the full three-dimensional vector space defined by the Stokes parameters (*S*_1_, *S*_2_, and *S*_3_). As such, it is able to map any polarization state on the Poincaré sphere, a necessary condition in order to perform polarimetry. Using this matrix and the measured intensities observed from arbitrary polarization states, any input polarization can be identified from the corresponding Stokes parameters. Figure 3c shows a good match between the Stokes parameters obtained from the measured intensities and those corresponding to the input polarization states.^22,24^ From the graphs and the extracted Stokes’ values, with this meta-polarimeter, we are able to resolve not only any linear polarization states but also elliptical polarizations and can determine any arbitrary polarization with a resolution of 5° (see Section S2 of the Supporting Information for more details). We calculated the fidelities between the polarization states measured using the meta-polarimeter  and the respective input polarization states  as . The input and measured states plotted on the Poincare sphere in Figure 3d give an average fidelity of *F* = 99.27 ± 0.86%. A clear difference in the intensities of various spots is seen in the diffraction patterns for the |*L*⟩/|*R*⟩ and elliptical polarizations as shown in Figure 3c and from the analysis in Figure 3d. The orientations of the Stokes parameters are shown in Figure 3e.

**Figure 4:**
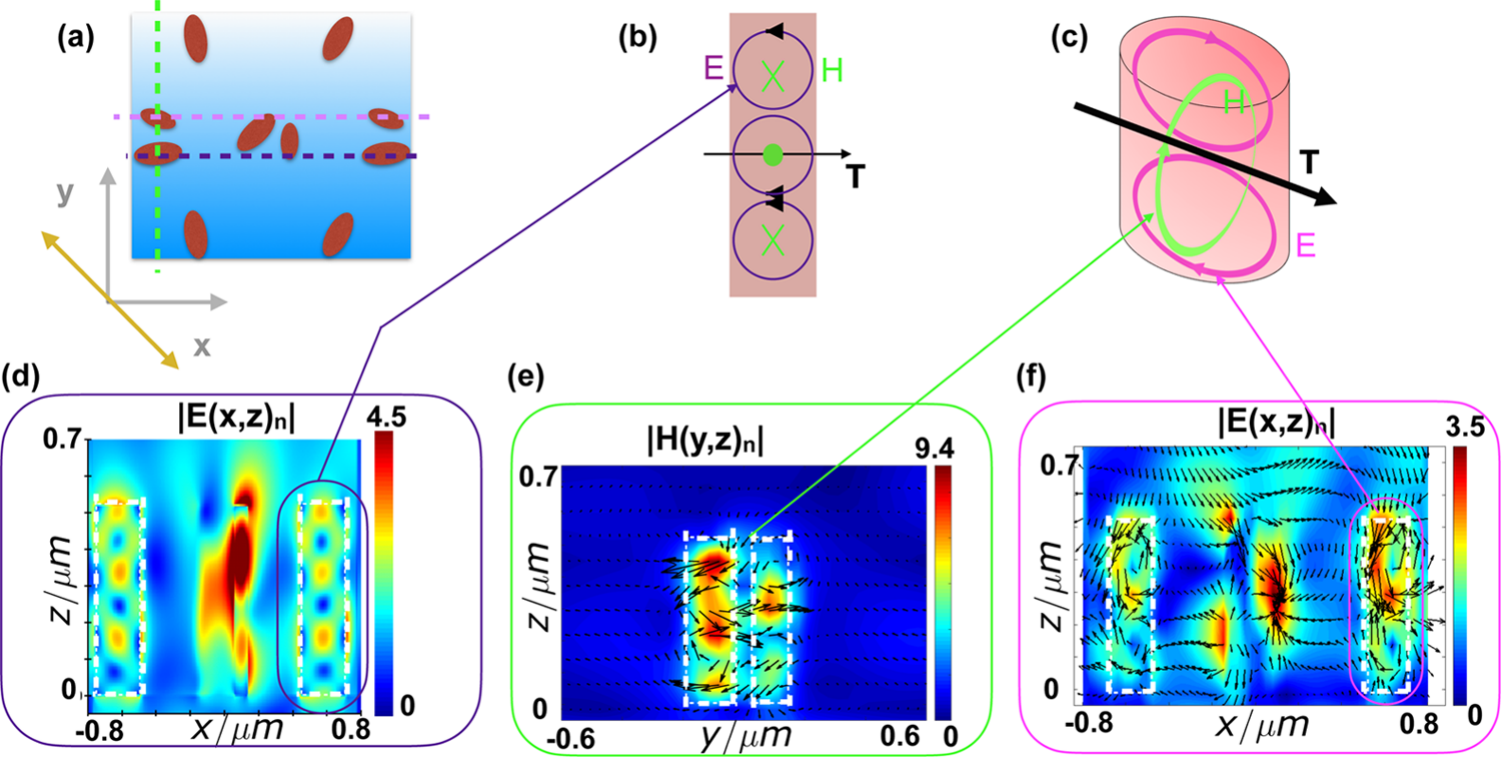
Normalized |*E*| and |*H*| fields of the bi-atom arrangements for |*A*⟩ polarization of incident light. (a) Illustration of the unit cell and its orientation. The dashed lines are guides to the eye, highlighting the relative positions of pairs of pillars as arranged within the meta-atom as well as the cross sections plotted below in (d,e,f), also indicated by the matching colors. (b) Illustration of the resonant modes. (c) Illustration of the anapole showing the coupling between the *E* and *H* fields. (d) Normalized *E*-field |*E*(*x*,*z*)_*n*_| for the larger meta-atom shown in the schematic. (e) Normalized |*H*(*y*,*z*)_*n*_| field and the vector field for the smaller meta-atom. (f) Signature of the formation of a non-radiating anapole state as observed from the simulated *E*-field (highlighted).

In terms of understanding the underlying physics, the polarization effect of a metasurface has been described by a Jones matrix that can be written in terms of the eigenmodes supported in the nanoresonator, Ψ_eig_^±^^17^
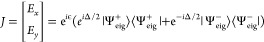
3where ϵ and Δ are the phase and phase retardation terms. Engineering the meta-atoms to excite different eigen-polarization states is the principal focus of this design. Given the asymmetric nature of the proposed design, we are not limited to bound states as is the case for the designs explored so far in the literature. Another degree of freedom exploited is that, for the bi-meta-atoms, the resonant modes^25,26^ occur in the pillars as well as the gap between the pillars. The different resonant eigenmodes that are excited by the different polarization states of the incident light are a combination of electric dipole, magnetic dipole, electric quadrupole, and magnetic octupole modes. The modes are a combination of Mie mode (surface) and the Fabry Perot mode formed within the pillar (open-ended oscillator approximation).^27−29^ The interplay between the vector moments of the various poles excited by the polarization of light results in a unique electric and magnetic (*E*–*H*) field patterns. Investigating the eigenmodes excited by the linearly polarized incident light, we analyzed the simulated normalized *E*–*H* fields of |*A*⟩ polarization state from the bi-meta-atom arrangement highlighted in the schematic in Figure 4. (Section S3 of the Supporting Information contains the *E*–*H* fields for |*H*⟩, |*V*⟩, |*A*⟩, and |*D*⟩ polarization states.)

Figure 4 shows the simulated |*E*(*x*,*z*)_*n*_| and |*H*(*y*,*z*)_*n*_| of the bi-meta-atoms’ arrangement as indicated in the schematic for |*A*⟩ polarized incident light (Figure 4a–c). The simulated fields in Figure 4d show *E*-field vortices. The induced current vector moments follow the electric field around the magnetic pole (Figure 4e) that circulates axially from head to tail. Similarly, in the smaller pillar, the magnetic dipole circulates axially through the current (*E*-field) vector moments. This leads to the formation of a toroidal moment (*T*) perpendicular to the *z* axis, as shown in the schematic in Figure 4b,c. The near-field radiation pattern from a toroidal pole and an electric dipole is identical,^30,31^ and the interaction between these poles form the non-radiative anapole. For the smaller elliptical pillar, we observe a dipole and a magnetic toroidal pole axially forming an anapole state. For the larger elliptical pillar, this combination of magnetic toroidal and electric multipoles forms a hybrid non-radiating anapole state (Figure 4f).

Due to the asymmetry of our structure, with wavelength fixed at 810 nm, the incident light of different polarizations excites different resonant modes in the nano structures. We give a qualitative analysis of the modes excited for the polarizations |*H*⟩, |*V*⟩, |*A*⟩, |*D*⟩, |*L*⟩, and |*R*⟩. For example, the distinct signature of the anapole state is shown in Figure 1 of ref^30^. With the vector plots in Figure 4f, we can gauge the moments of the electrical and magnetic dipole and confirm that it is an anapole state. Also, the lower intensity of the spots for |*A*⟩ polarization supports the hypothesis. For arbitrary polarizations, the modes in these pillars would be a combination of |*A*⟩ and |*D*⟩ states with varying weightings of the contributing modes (also see Section S3 in the Supporting Information for extended numerical simulation plots and details).

Given the different eigenmodes excited for the base linear orthogonal polarizations, the modes excited for |*L*⟩ and |*R*⟩ are different, which leads to the difference in the diffraction patterns (that can be extended to any elliptical polarization state). The modal differences are highlighted further in Section S3 of the Supporting Information.

We note that the 63.7% diffraction efficiency of our design is wavelength-specific and is for a relatively small bandwidth (typically ∼10 nm^31^) around the resonance frequency of the anapole state. While the PB phase approach is more broadband as it relies on the phase imparted to the incident light, a single-unit-cell design is unable to resolve all polarization states. In the literature, the ability to resolve between the six degenerate polarization states has been achieved by using a combination of three designs for the measurement in the {|*H*⟩, |*V*⟩}, {|*A*⟩, |*D*⟩}, and {|*R*⟩, and |*L*⟩} polarization bases, respectively. However, this also rotates the handedness of the elliptical/circular states, making it difficult to measure arbitrary polarization states. The trade-off is that our design allows for the measurement of arbitrary polarizations since it is the diffraction pattern that is unique for arbitrary polarization states rather than having a given degenerate polarization state deflected to a specific spot. We also note that holographic approaches, although using PB phase designs, are wavelength-specific as well.

### Conclusions

In conclusion, we demonstrate a dielectric meta-polarimeter that provides a unique diffraction pattern for any arbitrary polarization state on the Poincaré sphere. The Stokes parameters were calculated from the intensities of four spots in the diffraction pattern, yielding an experimental measurement fidelity of up to 99%. The formation of the anapole state makes it possible to detect not just linear but elliptical polarization states with the same design. With this single optical component, we achieved polarimetry measurements out-performing existing polarimetric techniques in terms of size and complexity. Although we have focused here on an experimental demonstration with pure polarization states (by using pure input states and normalized Stokes vectors), general polarization states—including mixed states—could be measured, for example, by using a maximum likelihood approach for determination of the full density matrix as done in ref^6^, corresponding to the determination of the full set of Stokes parameters. This could be useful, for example, for quantifying the degree of polarization of light or for full quantum tomography of polarization states.

## Methods

### Fabrication

The substrate was a borosilicate glass slide with the dimensions of 20 mm × 20 mm and a thickness of 515 μm. 520 ± 5 nm of amorphous Silicon (α-Si) was deposited using plasma-enhanced chemical vapor deposition on the borosilicate substrate that was attached to a carrier wafer (with a layer of SiO_2_). The samples were cleaned with acetone and isopropyl alcohol (IPA) in an ultrasonic bath. The e-beam resist (all-resist PMMA 632.06 50K) was spin-coated at 4000 rpm for 60 s and baked in the oven at 180 °C for 30 min. This was followed by spin-coating a second layer of e-beam resist (all resist PMMA 679.02 950K) at 4000 rpm for 60 s followed by baking it in the oven at 180 °C for 60 min. A 20 nm layer of Al was deposited using the e-beam evaporator Plassys II, which acts as a charge conduction layer for the e-beam writing. The exposed patterns were immersed in a solution of MFCD-26 for 2 min to remove this charge conduction layer. After thoroughly rinsing in IPA and blow drying with an N2 gun, the samples were developed in a 2:1 ratio of IPA/MIBK for 30 s at 23 °C, followed by a rinse in IPA for 30 s. Plasma ashing with oxygen in the Oxford Instruments Plasmalab 80 Plus reactive ion etcher was done to remove any developed residue. This was followed by a metallization step: 50 nm of NiCr deposited by the e-beam evaporator, which acts as a hard mask for etching. The samples were placed in an acetone bath maintained at 50 °C for lift-off. The samples were etched with a C4F8/SF6 chemistry in STS ICP followed by cleaning in acetone, IPA and HMDSO (without ultrasonication). The NiCr layer was removed using a chrome etchant followed by nitric acid (to remove the Ni layer). Finally, the samples were cleaned in acetone and IPA and imaged with the FEI Nova NanoSEM 630 scanning electron microscope.

### Simulation Setup

The amorphous silicon pillars defined in the unit cell were placed on a fused silica substrate. The material parameters for α-Si were measured with the ellipsometer Bruker in the James Watt Nanofabrication Center (JWNC, University of Glasgow) clean room and the refractive index information and used in the simulations. For transmission simulation of the far-field diffracted spots, periodic boundary conditions were used around the super cell (shown in Figure 1a). A mesh grid with a maximum cell size of 5 nm was defined in the vicinity of the nanopillars and the interface with the substrate. Steep angle boundary conditions were used in the FDTD simulation to absorb all the light at the boundaries and prevent any spurious reflections. The metasurface was illuminated by a 808 nm plane-wave source from within the substrate, and the transmission spectra were recorded with a monitor placed on the opposite side of the nanopillars. A total-field scattering-field source was used for determination of the scattered field from the nanopillars. To obtain the *E* and *H* vector fields for the various polarization states, a cell area was considered with perfectly matched layers boundary conditions in the *x*, *y*, and *z* axes. The FDTD boundaries were several orders of magnitude greater than the wavelength λ. The stability factor was set to 0.7 in order to achieve simulation convergence.

### Measurement Setup

For the single shot imaging from the metasurface polarimetry, the measurement setup is shown in Figure 1d. A Coherent Chameleon Discovery laser at 808 nm was coupled into a fiber since ∼808 nm is the calculated resonance wavelength of the anapole state. To set the input polarization state onto the metasurface, a polarizing beam splitter (PBS), HWP and QWP was used. To focus light onto the metasurface, a focusing lens was used after the PBS such that the beam waist was smaller than the metasurface area. For the collection optics of the diffraction pattern from the metasurface, a combination of a CL and 2″ FL was used. The CL was positioned to collect all the diffraction spots and collimate the beams of light, which were, in turn, collected using the FL and focused onto a camera. All images were collected with an Andor Marana sCMOS camera with an integration time of 7 ms.

## Supporting Information Available

Experimental results and simulations that complement the main results of the manuscript; lumerical FDTD simulations that show the design principle used; diffraction patterns obtained experimentally compared with simulations; repeatability from another fabricated device; simulated *E*–*H* fields for |*H*⟩, |*V*⟩, |*A*⟩, and |*D*⟩ polarization states; and detailed *E*–*H* fields that show different modal resonances in |*R*⟩ and |*L*⟩ states (PDF)

## Notes

Data used in this manuscript are available from DOI: http://dx.doi.org/10.5525/gla.researchdata.1347.
