# Supplementary material for: An All-Dielectric Metasurface Polarimeter
Source: ACS Photonics. 2022 Sep 15;9(10):3245–52. doi: 10.1021/acsphotonics.2c00395 (PMC9585641; doi:10.1021/acsphotonics.2c00395)
Supplement: Supplementary file 1 — ph2c00395_si_001.pdf [file ph2c00395_si_001.pdf]

Supplementary information for "An all-dielectric metasurface polarimeter"

Yash D. Shah,[†,§] Adetunmise C. Dada,[†,§] James P. Grant,[‡] David R. S. Cumming,[‡] Charles Altuzarra,[†] Thomas S. Nowack,[‡] Ashley Lyons,[†] Matteo Clerici,[¶] and Daniele Faccio[*,†]

†*School of Physics and Astronomy, University of Glasgow, Glasgow, UK*

‡*Microsystems Technology Group, James Watt School of Engineering, University of Glasgow, Glasgow, UK.*

¶*James Watt School of Engineering, University of Glasgow, Glasgow, UK.*

§*These authors contributed equally to this work.*

E-mail: Daniele.Faccio@glasgow.ac.uk

This supplementary information contains simulations and experimental results that complement the main manuscript text.

S1. Simulations for metasurface design

The geometry and orientation of the meta-atoms of the unit cell were varied until we obtained the best trade-off for the design principle: 1) highest diffraction efficiency and 2) highest difference in intensity between $|R\rangle$ and $|L\rangle$ incident polarization state (i.e., left- and right-circular polarization states, respectively). We start off with Lumerical finite-difference time-domain (FDTD) simulations on the height of the nanopillars. The shaded region in Figure S1 (a) shows the ideal range in which the highest transmission efficiency (63.7%) was obtained.

For the bi-meta-atom arrangement, as noted in Refs 1 and 2, a small change in the symmetry creates leaky quasi bound-in-continuum (BIC) states. Using the dimensions and orientation of the bi-atom arrangement, we are able to tune the resonant modes through polarization of light. In Ref. 3, a bi-atom arrangement that maintained symmetry along the y-axis would in principle excite the same modes for both $|L\rangle$ and $|R\rangle$ states, as there is no asymmetry in the linear polarized state to break the symmetry dependence. So α is the symmetry parameter in the geometry, that is how the one of the bi atoms is scaled to the other, as shown in Figure S1 (b). Using the values in the window highlighted, the angle between the meta-atoms (Ξ) was varied and Δ_{LR} was calculated from simulation results and plotted in Figure S1 (c). Finally, the orientation of the entire bi-meta-atom, Γ, was varied to obtain the highest Δ_{LR}.

S2. Measurements

The diffraction patterns for the orthogonal basis states and the simulated results are shown in Figure S2. To highlight the repeatability, we show the diffraction pattern for the same polarization states from another device in Figure S3. In the main text, we quantify the diffraction pattern by taking the difference of the diffraction pattern from an arbitrary polarization state and the combined intensities of $|H\rangle$ and $|V\rangle$ polarization states. This is shown in Figure S4 where ΔI_D^{HV} is plotted. Given the spread of the diffraction spot, we take the probability density function along the x and y axis. This is shown in the inset in Figure S4.

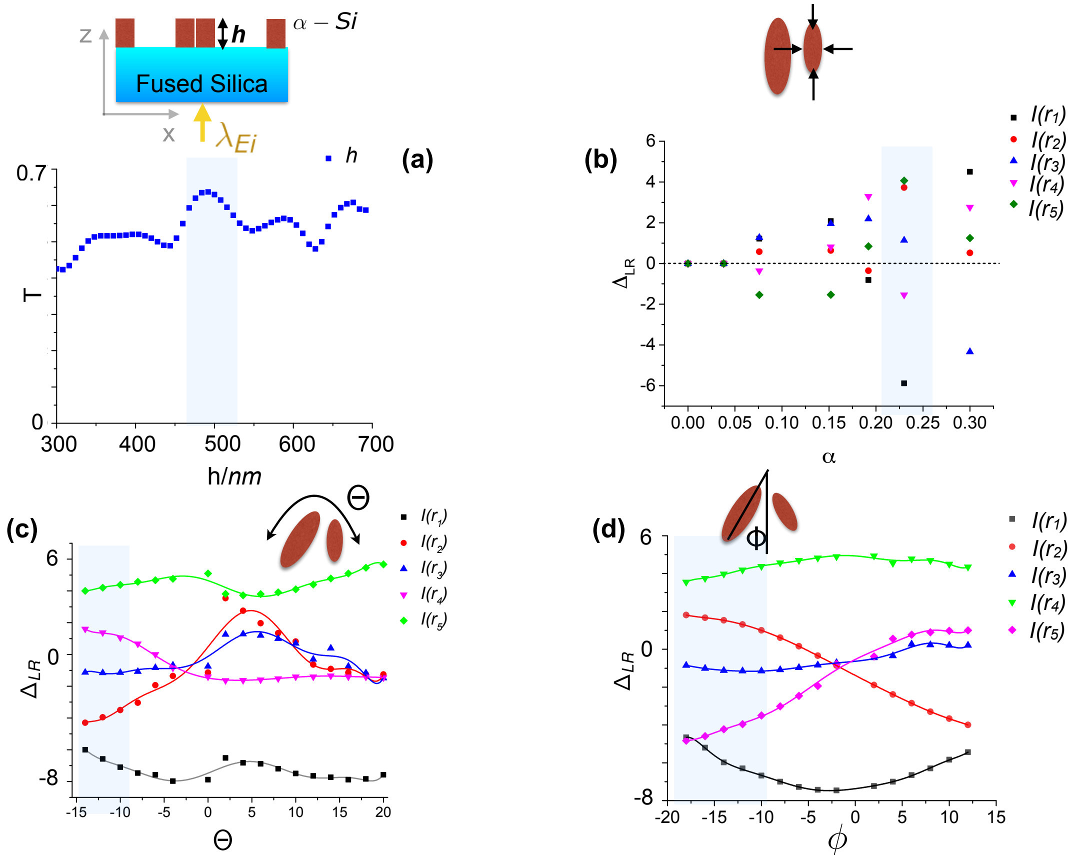

Figure S1: (A) Lumerical FDTD simulations for height h of the metasurface nanopillars. The height giving the highest transmission was within the range 485-525 nm. (B) Using design principles 1 and 2 for the asymmetry in geometry, the difference Δ_{LR} for the diffracted spots is shown and an ideal value of 0.25 is chosen. (C) Δ_{LR} as a function of the orientation of the elements (D) Δ_{LR} as a function of the orientation of the bi-atom.

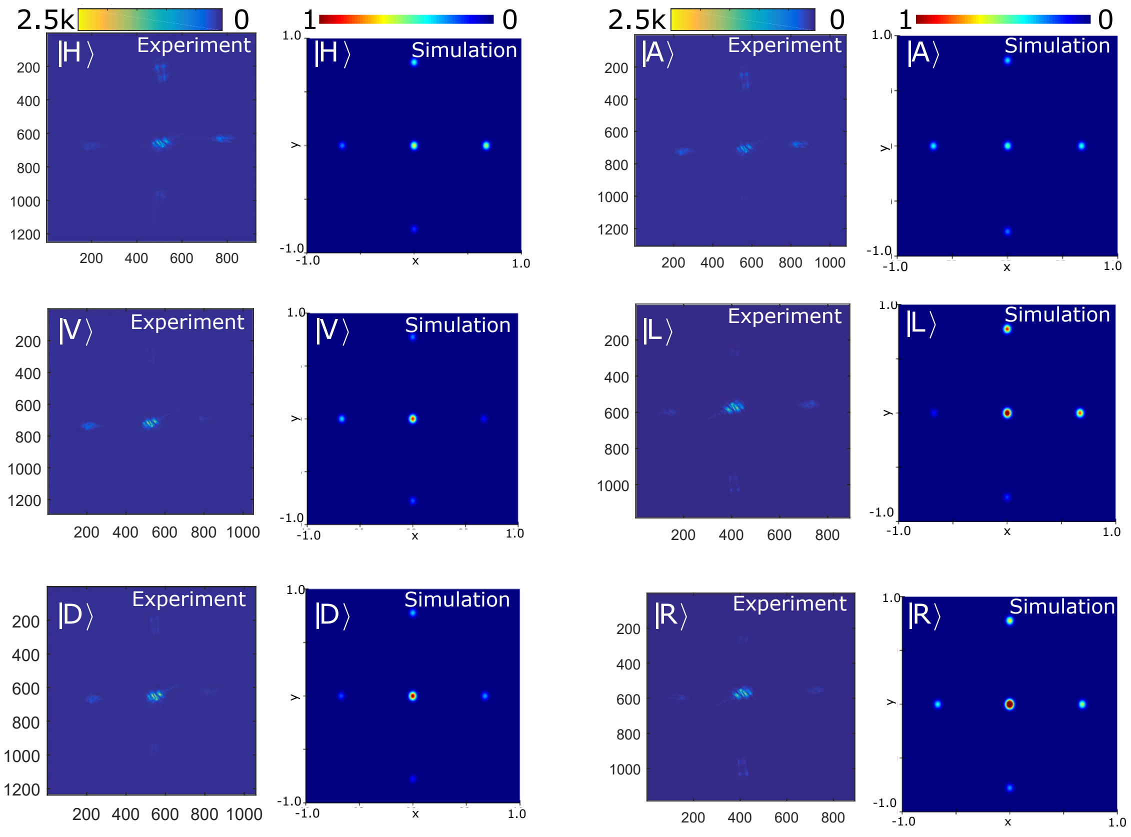

Figure S2: Diffraction pattern from a metasurface polarimeter along with the simulated results for $|H\rangle$, $|V\rangle$, $|D\rangle$, $|A\rangle$, $|L\rangle$ and $|R\rangle$ states respectively.

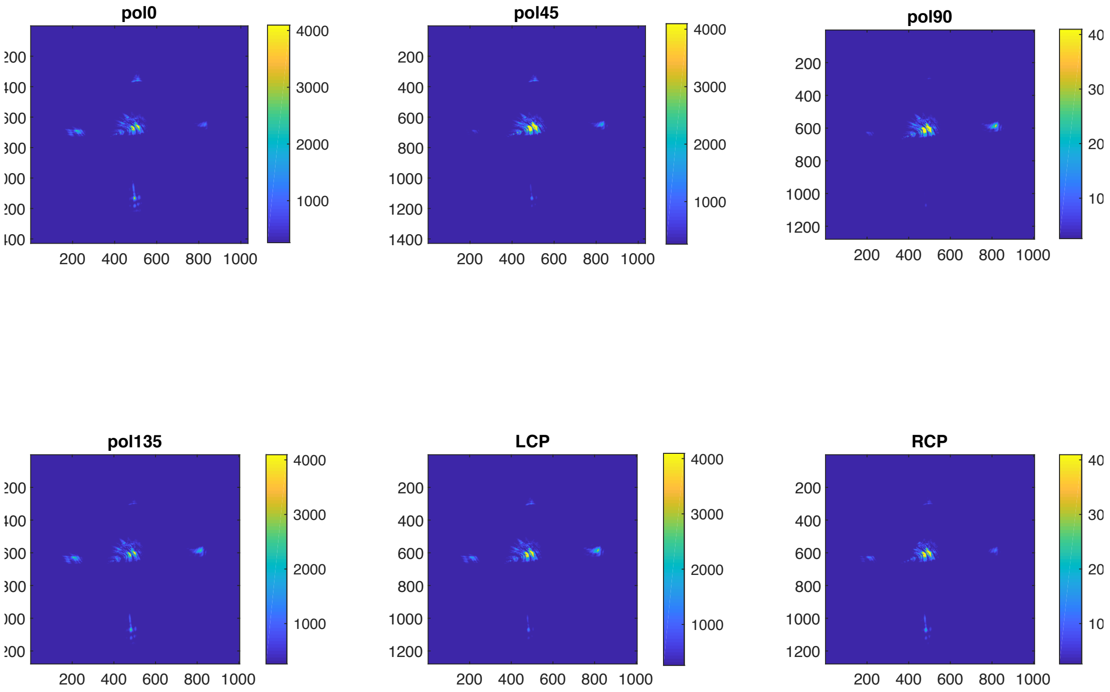

Figure S3: Test of reproducibility by showing the diffraction pattern from another metasurface device.

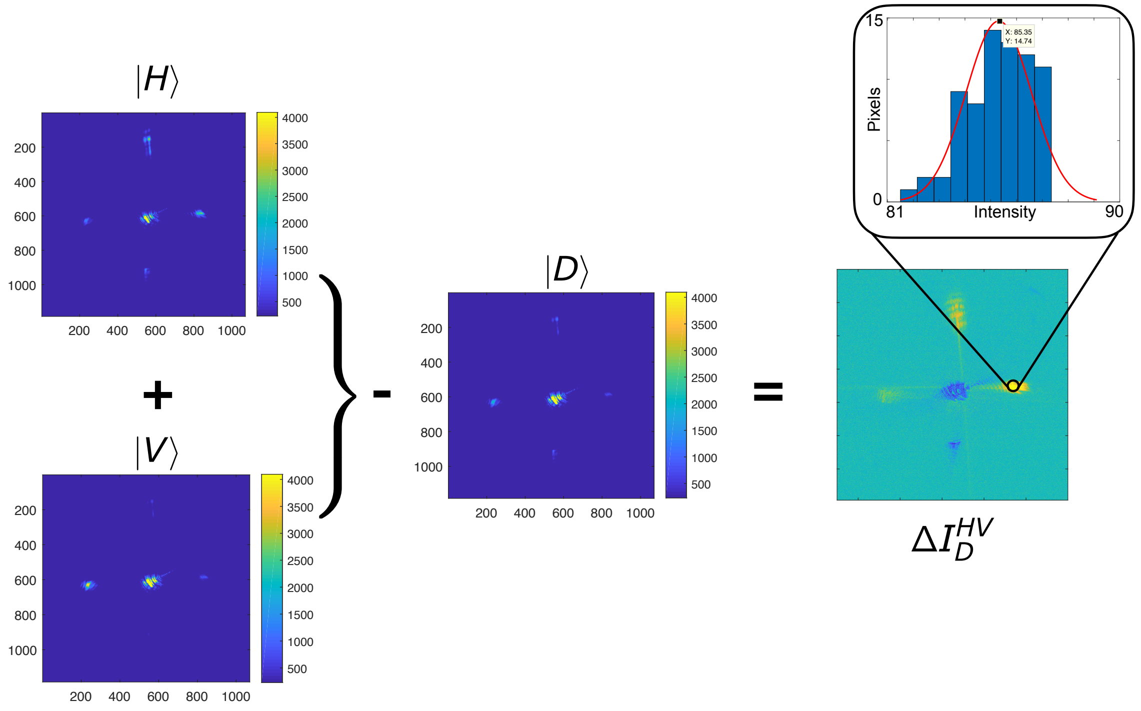

Figure S4: The intensity difference patterns (ΔI_D^{HV}) were calculated as shown for polarization state $|D\rangle$ with respect to the combined intensities of $|H\rangle$ and $|V\rangle$. The probability distribution was obtained in 2 dimensions to get the exact value for every diffracted spot.

S3. E-H fields from the meta-atoms for orthogonal basis polarization states

Figure S5 shows the simulated $|E(x,z)_n|$ and $|H(y,z)_n|$ of the bi-meta-atoms arrangement as indicated in the schematic for $|H\rangle$, $|V\rangle$, $|A\rangle$ and $|D\rangle$ polarization states. For the incident $|V\rangle$ polarization state, the E-field shows an electrical resonance from the mode confined between the pillars with no excited eigenmodes within the larger pillar and a TM_{21} mode oriented along the larger axis of the smaller elliptical pillar. Similarly analyzing the E-H fields for polarization $|H\rangle$ we observe a magnetic resonance in the larger elliptical pillar [TM_{21} mode in Figure S5 (d)] and an electrical resonance in the smaller elliptical pillar [Figure S5 (e)]. We observe an anapole state for the $|A\rangle$ polarization state and no anapole state signature in the E-field for the $|D\rangle$ polarization state. For the $|D\rangle$ polarization state: we observe a magnetic resonance in the larger pillar [inferred from electrical vortex in Figure S5 (d)] and

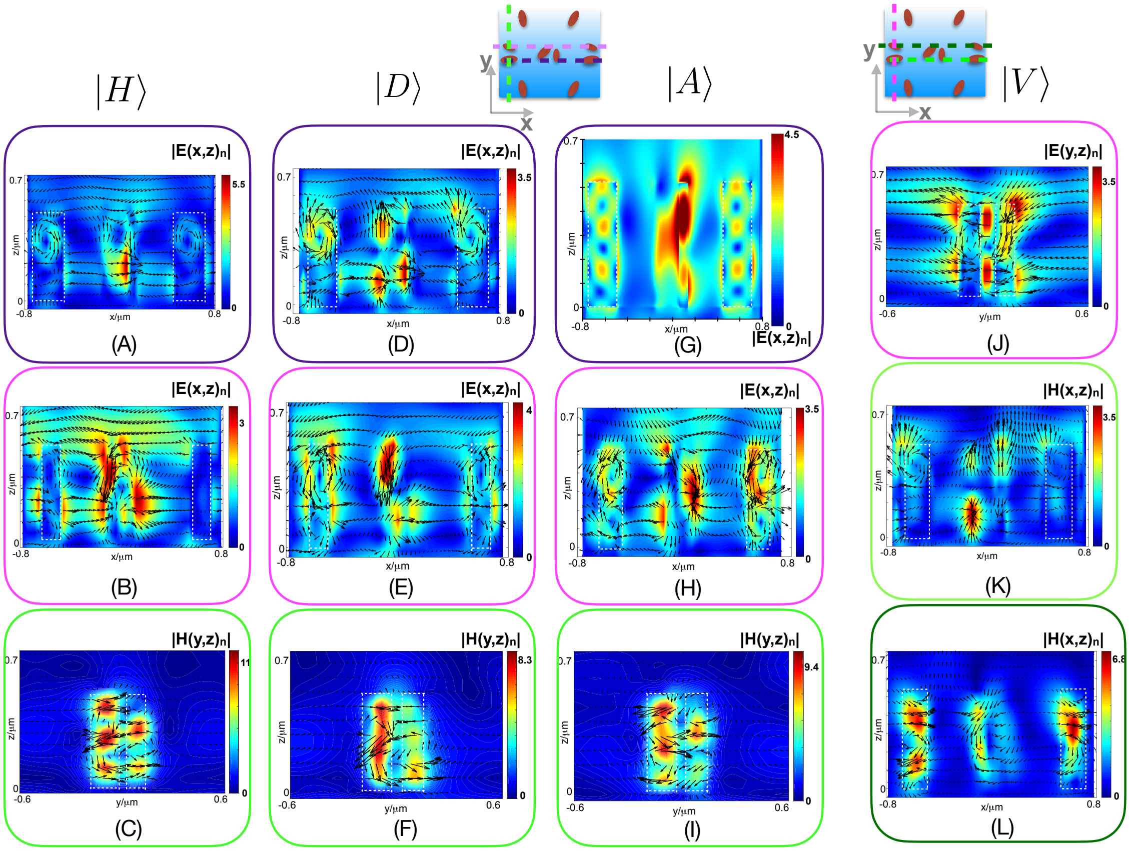

Figure S5: $|\mathbf{E}|$ and $|\mathbf{H}|$ field profiles normalized to the intensity of the incident light under $|H\rangle$, $|V\rangle$, $|A\rangle$ and $|D\rangle$ polarizations. The $|\mathbf{H}|$ fields are in green and the $|\mathbf{E}|$ fields in purple and pink.

we do not observe an electrical dipole in the smaller pillar [Figure S5 (e)].

Figure S6 (a) shows the $|\mathbf{H}(y,z)n|$ field distribution for $|L\rangle$ polarized incident light wherein a more axial field is observed indicating a stronger Mie resonance than Fabry Pérot mode. This is significantly different from with the **H**-field for $|R\rangle$ polarized incident light which shows a stronger Fabry Pérot mode, with a magnetic octupole (MO) and a magnetic dipole (MD) in the larger and smaller pillars respectively. Although the E-field profiles in the larger pillar is similar for both polarizations as seen in Figures S6 (b) and (c) the smaller pillar for $|L\rangle$ polarized light shows a modal interaction between a weak TM_{21} and TE_{12} mode whereas for $|R\rangle$ polarized light no resonant modes in the same pillar is observed. Since the E and H field profiles are unchanged in the extreme lattice points (single meta-atoms) for $|L\rangle$ and $|R\rangle$ polarized light, we can conclude that the difference in circular as well as elliptical polarized light are due to the different modes that couple with the incident in the bi-meta-atom arrangement.

Figure S7 (a) (i) shows the difference in intensity of the diffraction spots between $|L\rangle$ and $|R\rangle$ polarization states, with the simulation results in (ii). Figure S7 (b) is the normalized H-field, $|H(x,z)n|$ for $|L\rangle$ polarized incident light. For this polarization of light, the simulations show a mix of a magnetic resonance and a magnetic vortex formed between the two pillars near the base. This is supported by the electrical resonance observed in Figure S7 (f) near the base which might suggest reflection. A radiative mode is seen in with a stronger TM_{21} mode mixed with a weaker TE_{12} mode as shown in Figure S7 (e). On the other hand for $|R\rangle$ polarized light from the $|\mathbf{H}(x,z)n|$ field a magnetic dipole resonance between the pillars is observed as shown in Figure S7 (c). Unlike $|L\rangle$ polarized light, an electrical resonance in the larger nano pillar [Figure S7 (g)] and a weaker E-field vortex is observed in the smaller nano pillar [Figure S7 (h)].

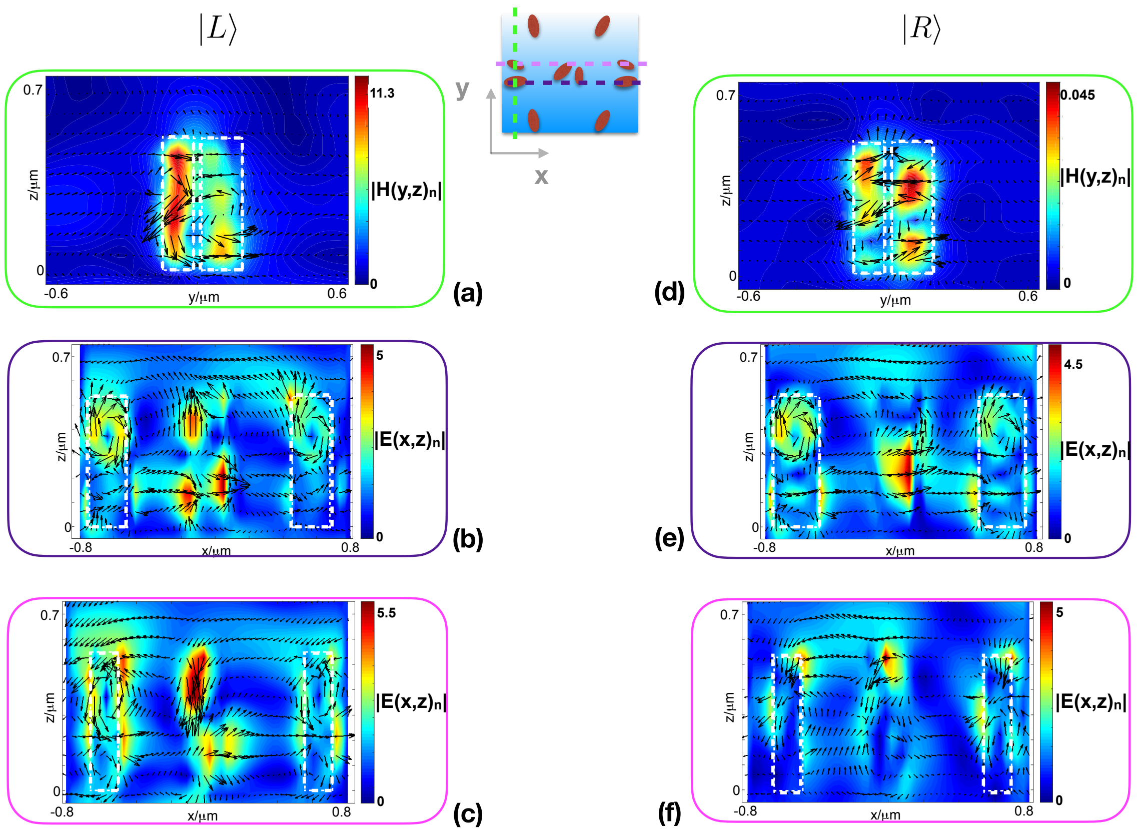

Figure S6: $|\mathbf{E}|$ and $|\mathbf{H}|$ field profiles normalized to the intensity of the incident light under $|R\rangle$ and $|L\rangle$ polarizations. The $|\mathbf{H}|$ fields are in green and the $|\mathbf{E}|$ fields in purple and pink.

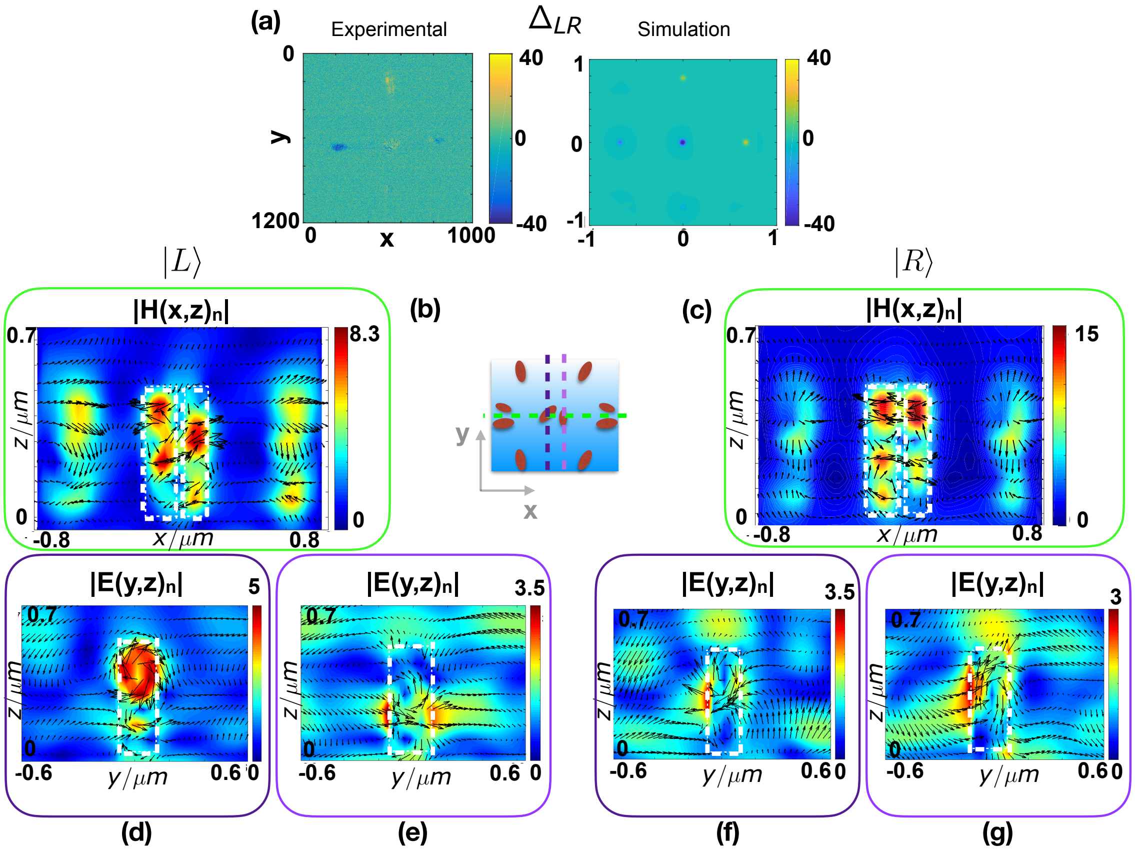

Figure S7: $|E|$ and $|H|$ field profiles normalized to the intensity of the incident light under $|R\rangle$ and $|L\rangle$ polarizations. The $|H|$ fields are in green and the $|E|$ fields in purple and pink. (A) Intensity difference, Δ_{LR}, between $|L\rangle$ and $|R\rangle$ polarizations for each measured diffracted spot plotted against the simulated intensity difference. (B) and (C) The magnetic field $|H(x, z)_n|$ for $|L\rangle$ and $|R\rangle$ polarization states, respectively. The electrical fields are shown in (E) and (F) for $|L\rangle$ polarization and, (G) and (H) for $|R\rangle$ polarization. Due to both these radiative modes the central spot has a higher intensity for $|R\rangle$ polarized light then $|L\rangle$ polarized light.

S4. Expansion on Figure 4 of the main text

References

(1) Leitis, A.; Tittl, A.; Liu, M.; Lee, B. H.; Gu, M. B.; Kivshar, Y. S.; Altug, H. Angle-multiplexed all-dielectric metasurfaces for broadband molecular fingerprint retrieval. *Science Advances* **2019**, *5(5)*, eaaw2871.

(2) Koshelev, K.; Lepeshov, S.; Liu, M.; Bogdanov, A.; Kivshar, Y. Asymmetric Metasurfaces with High- Q Resonances Governed by Bound States in the Continuum. *Physical Review Letters* **2018**, *121*, 193903.

(3) Liu, M.; Choi, D. Y. Extreme Huygens' Metasurfaces Based on Quasi-Bound States in the Continuum. *Nano Letters* **2018**, *18*, 8062–8069.
